# National Patterns in Environmental Injustice and Inequality: Outdoor NO_2_ Air Pollution in the United States

**DOI:** 10.1371/journal.pone.0094431

**Published:** 2014-04-15

**Authors:** Lara P. Clark, Dylan B. Millet, Julian D. Marshall

**Affiliations:** 1 Department of Civil Engineering, University of Minnesota, Minneapolis, Minnesota, United States of America; 2 Department of Soil, Water and Climate, University of Minnesota, Minneapolis, Minnesota, United States of America; Tsinghua University, China

## Abstract

We describe spatial patterns in environmental injustice and inequality for residential outdoor nitrogen dioxide (NO_2_) concentrations in the contiguous United States. Our approach employs Census demographic data and a recently published high-resolution dataset of outdoor NO_2_ concentrations. Nationally, population-weighted mean NO_2_ concentrations are 4.6 ppb (38%, *p*<0.01) higher for nonwhites than for whites. The environmental health implications of that concentration disparity are compelling. For example, we estimate that reducing nonwhites’ NO_2_ concentrations to levels experienced by whites would reduce Ischemic Heart Disease (IHD) mortality by ∼7,000 deaths per year, which is equivalent to 16 million people increasing their physical activity level from inactive (0 hours/week of physical activity) to sufficiently active (>2.5 hours/week of physical activity). Inequality for NO_2_ concentration is greater than inequality for income (Atkinson Index: 0.11 versus 0.08). Low-income nonwhite young children and elderly people are disproportionately exposed to residential outdoor NO_2_. Our findings establish a national context for previous work that has documented air pollution environmental injustice and inequality within individual US metropolitan areas and regions. Results given here can aid policy-makers in identifying locations with high environmental injustice and inequality. For example, states with both high injustice and high inequality (top quintile) for outdoor residential NO_2_ include New York, Michigan, and Wisconsin.

## Introduction

Environmental injustice often places disproportionate health risks on people who are already the most vulnerable or susceptible to those risks. Since the earliest US environmental justice studies [1]–[6] in the 1960s–1980s, disparities in exposures to environmental risks (e.g., landfills, hazardous waste sites, polluting industries, vehicle traffic) by socioeconomic status (SES) have been widely documented [7]–[9]. Air pollution is a priority environmental risk in the United States (US): urban outdoor air pollution is one of the top ten causes of death in high-income nations [10]. Low-SES communities are often disproportionately exposed to air pollution [11] and also may be more susceptible to air pollution owing to other underlying disparities in, for example, access to health care [12].

Although relationships between air pollution exposure and SES have been documented in certain US cities, little is known about the broader patterns in ambient air pollution environmental justice within and across US geographies (cities, regions, states, urban versus rural areas). This previous lack of understanding is largely because of the limited coverage and spatial resolution of ambient air pollution data. Recent work exploring air pollution environmental justice in US cities or regions has been based on industrial emissions-based air pollution concentration estimates [13]–[16], or has focused on people living near regulatory monitor locations [17]–[19]. Those multi-city and national studies reported differences in environmental injustice by US region [18], metropolitan area [13] and urban form characteristics of metropolitan areas [15]–[17].

Here, we employ a recently developed ambient air pollution dataset [20] to explore patterns in environmental justice within and across US geographies, including rural and urban populations. The work applies a national land use regression with high spatial resolution (∼0.1 km) to examine residential outdoor nitrogen dioxide (NO_2_) air pollution in the US. NO_2_, which is one of the six US Environmental Protection Agency criteria pollutants, in the US is mainly emitted (as NO_x_) from combustion in vehicles and power plants [21]; it is a marker for traffic emissions [22] and has high within-urban variability [23], [24]. NO_2_ and other traffic emissions are linked to asthma [25] and decreased lung function [26] in children, low birth-weights [27], and cardiovascular and respiratory mortality (e.g., ischemic heart disease mortality) [28], [29]. Previous work in specific US cities suggests that ambient NO_2_ (and/or NO_x_) concentrations tend to be higher in low- than in high-SES communities [30]–[33].

This paper applies a national-scale analysis to quantify US-wide NO_2_ concentration patterns by SES characteristics. It provides quantitative information for understanding how environmental equality and justice for air pollution vary among communities and regions across the US. A goal of this study is to identify US locations with highest priority environmental justice and equality concerns attributable to NO_2_ and co-emitted air pollutants.

## Methods

### 1. Data

Our analysis covers the year-2000 population of the contiguous US (280 million people). The spatial unit of analysis is the Census Block Group (BG), which is the smallest Census geography with demographic data (race-ethnicity, household income, poverty status, education status, and age) reported in the 2000 Census. Of all BGs (*n* = 207,492), 64% are urban, 14% are rural, and 21% are mixed urban-rural (i.e., contain both urban and rural Census Blocks). The mean BG sizes are 1.1 km^2^ (urban), 185 km^2^ (rural), and 45 km^2^ (mixed); the mean (standard deviation) BG population is 1,350 (890) people.

Air pollution data are year-2006 annual average ground-level NO_2_ concentration estimates from a recently published national land use regression (LUR) [20]. This LUR predicts NO_2_ concentrations at the Census Block level for the contiguous US based on satellite- and ground-based measurements of NO_2_, combined with land use data (e.g., road locations, elevation, tree cover, impervious-surface coverage, population density). To match the Census BG level demographic data, we calculate the mean concentration among all Blocks in each BG. Nationally, the mean NO_2_ concentration for all BGs is 11.4 ppb.

### 2. Statistical Analyses

We calculate population-weighted mean NO_2_ concentrations by race-ethnicity, poverty status, household income, education status, and age, using annual mean BG concentrations (from year-2006 LUR data) and population estimates (from year-2000 Census data). For example, the national population-weighted mean NO_2_ concentration for nonwhites is the mean of BG mean concentrations weighted by the population of nonwhites in each BG. We then calculate environmental injustice and inequality metrics by US region, state, county, and Urban Area (UA), and rural versus urban location.

Our primary comparison metric for environmental injustice is the difference (ppb) in population-weighted mean NO_2_ concentration between lower-income nonwhites (LIN; nonwhites in the lowest annual household income quintile [<$20,000]) and higher-income whites (HIW; whites in the highest annual household income quintile [>$75,000]). Our primary comparison metric for environmental inequality is the Atkinson Index (ε = 0.75 [34]–[38]), which measures the extent to which NO_2_ concentrations are evenly distributed across the population: Atkinson Index = 0 indicates perfect equality (i.e., concentrations are equal for all people); higher values indicate greater inequality (maximum = 1). The US Census information about race covers 100% of the population, whereas combined race-income categories (e.g., whites with income >$75,000) are only available for 38% of the population (one person per household; “householders”). Our injustice metric includes 10% of the total Census population (26% of householders): lower-income nonwhite householders are 2.9% of the total Census population; higher-income white householders are 7.0%. In contrast, the inequality metric and straightforward white/nonwhite comparisons include 100% of the total Census population. See Supporting Information (**Figures S1–S2** and **Table S1** in **File S1**) for sensitivity analyses regarding metric selection.

## Results and Discussion

Our results reveal significant disparities in NO_2_ concentrations for specific socioeconomic groups (**Table 1** **;** **Table 2**). For example, average NO_2_ concentrations are 4.6 ppb (38%, *p*<0.01) higher for nonwhites than for whites, 1.2 ppb (10%, *p*<0.01) higher for people below versus above poverty level, and 3.4 ppb (27%, *p*<0.01) higher for lower-income nonwhites than for higher-income whites. Likewise, NO_2_ concentrations are higher for residents with less than a high school education compared to those with a high school education or above (difference: 0.9 ppb [8%], *p*<0.01). Among urban residents, NO_2_ concentrations for Black Hispanics (the most exposed race-ethnicity group) are 6.1 ppb (38%, *p*<0.01) higher than for American Indians (the least exposed race-ethnicity group) and 4.7 ppb (28%, *p*<0.01) higher than for the total urban population. Urban-rural differences abound: in urban areas, NO_2_ concentrations are higher for nonwhites than for whites, and higher for low- than for high-income groups; in contrast, NO_2_ concentrations in rural areas are similar for nonwhites and for whites but are slightly lower for low- than for high-income groups. Urban areas exhibit more low- than high-income communities in NO_2_-polluted areas (e.g., adjacent to busy roadways), whereas the same trend does not emerge in rural areas. Among race-ethnicity groups, American Indians have the lowest NO_2_ exposures in urban areas, but the second highest NO_2_ exposures (after Hispanics) in rural areas. Overall, for seven of the eight nonwhite race-ethnicity groups considered (upper portion of **Table 1**), NO_2_ concentrations are higher for that group than for whites.

**Table 1:** Population-weighted mean NO_2_ concentration in ppb (percent of total population*1*).

	Total	Urban	Mixed	Rural
*Total*	11.3 (100%)	14.2 (63%)	7.3 (25%)	4.4 (12%)
*Race-ethnicity* 2				
White	9.9 (69%)	12.9 (38%)	7.1 (20%)	4.4 (11%)
Nonwhite	14.5 (31%)	16.4 (24%)	8.1 (4.6%)	4.5 (1.6%)
Hispanic	15.6 (13%)	17.2 (10%)	8.6 (1.8%)	5.8 (0.4%)
Black	13.3 (12%)	15.3 (9.4%)	7.4 (1.9%)	3.7 (0.8%)
Asian	16.5 (3.4%)	17.5 (3.0%)	9.7 (0.4%)	4.8 (0.03%)
Two or more races	13.1 (1.6%)	15.3 (1.2%)	7.9 (0.3%)	4.5 (0.1%)
Amer. Indian/Alaska Native	8.8 (0.7%)	12.8 (0.3%)	7.2 (0.2%)	5.4 (0.2%)
Black Hispanic	17.4 (0.3%)	18.9 (0.2%)	9.0 (0.03%)	4.2 (0.01%)
Other race	15.0 (0.2%)	16.9 (0.1%)	8.3 (0.03%)	4.7 (0.01%)
Nat. Hawaiian/Pacific Islander	14.2 (0.1%)	15.7 (0.1%)	8.4 (0.01%)	4.7 (0.003%)
*Poverty status*				
Below poverty level	12.4 (12%)	15.3 (8.2%)	7.3 (2.3%)	4.3 (1.5%)
Above poverty level	11.2 (85%)	14.1 (53%)	7.3 (22%)	4.5 (10%)
*Household income quintile*				
<$20,000	11.4 (8.3%)	14.4 (5.3%)	7.3 (1.8%)	4.3 (1.2%)
$20,000–$35,000	11.0 (7.3%)	13.9 (4.6%)	7.2 (1.7%)	4.4 (1.0%)
$35,000–$50,000	10.9 (6.2%)	13.9 (3.8%)	7.2 (1.5%)	4.4 (0.8%)
$50,000–$75,000	11.0 (7.3%)	13.9 (4.5%)	7.3 (1.9%)	4.5 (0.9%)
>$75,000	11.7 (8.4%)	14.2 (5.5%)	7.7 (2.3%)	4.6 (0.6%)
*Education level for population >25 years old*				
Less than high school degree	12.0 (13%)	15.5 (8.0%)	7.2 (2.8%)	4.3 (1.9%)
High school degree	10.5 (19%)	13.9 (10%)	7.1 (5.0%)	4.4 (3.1%)
Some post-secondary	11.0 (18%)	13.8 (11%)	7.3 (4.6%)	4.5 (2.0%)
Bachelor’s degree	11.7 (10%)	14.0 (6.8%)	7.6 (2.5%)	4.5 (0.7%)
Graduate degree	12.1 (5.7%)	14.3 (4.0%)	7.7 (1.4%)	4.5 (0.4%)
*Age*				
<5 years	11.6 (6.8%)	14.4 (4.4%)	7.4 (1.7%)	4.5 (0.8%)
5 to 18 years	11.2 (19%)	14.2 (12%)	7.2 (4.8%)	4.5 (2.4%)
18 to 40 years	11.8 (32%)	14.5 (21%)	7.4 (7.4%)	4.4 (3.3%)
40 to 65 years	11.0 (30%)	14.1 (18%)	7.2 (7.9%)	4.4 (4.0%)
>65 years	11.0 (12%)	13.9 (7.7%)	7.3 (3.1%)	4.4 (1.7%)
*Children (<5 years) below poverty level*				
White	9.1 (0.4%)	12.5 (0.2%)	6.9 (0.1%)	4.3 (0.1%)
Nonwhite	14.3 (0.8%)	16.1 (0.6%)	7.9 (0.1%)	4.7 (0.1%)
*Elderly (>65 years) below poverty level*				
White	9.9 (0.8%)	13.5 (0.4%)	7.1 (0.2%)	4.2 (0.2%)
Nonwhite	14.5 (0.2%)	16.9 (0.2%)	7.7 (0.03%)	4.3 (0.02%)

^*1*^Population totals may be less than 100% because of rounding, nonresponses in Census data, and category definitions (e.g., population >25 years old is 66% of total population).

^*2*^Each race-ethnicity category in **Table 1** includes people who reported a single race category and non-Hispanic ethnicity (i.e., “White” category is “White alone; non-Hispanic”), except for the “Hispanic” category, which includes people who reported any race(s) and Hispanic ethnicity, and the “Black Hispanic” category, which includes people who reported Black race alone and Hispanic ethnicity.

**Table 2:** Comparisons between population-weighted mean NO_2_ concentrations for specific populations.

Group 1 (concentration in ppb)	Group 2 (concentration in ppb)	Difference^1^ (ppb)	Relative Difference (%)
*National comparisons*			
Nonwhites (14.5)	Whites (9.9)	4.6	38
Below poverty (12.4)	At or above poverty (11.2)	1.2	10
Low-income nonwhites (14.4)	High-income whites (11.0)	3.4	27
Less than high school degree (12.0)	High school degree or above (11.1)	0.9	8
Children<5 years (11.6)	Age 5 to 65 years (11.3)	0.2	2
Nonwhite children below poverty level (14.3)poverty	Age 5 to 65 years (11.3)	3.0	23
Elderly>65 years (11.0)	Age 5 to 65 years (11.3)	−0.3	−3
Nonwhite elderly below poverty level (14.5)	Age 5 to 65 years (11.3)	3.1	24
*Urban comparisons*			
Black Hispanics (18.9)	American Indians (12.8)	6.1	38
Black Hispanics (18.9)	Total (14.2)	4.7	28

^*1*^Difference in population-weighted mean concentration [Group 1 - Group 2]. For all rows, differences are statistically significant with *p*<0.001.

Young children and the elderly are especially vulnerable to air pollution. We find that NO_2_ concentrations for these groups correlate with SES. Population-weighted mean NO_2_ concentrations are similar (within 3% [0.3 ppb]) for those two subpopulations (elderly: greater than 65 years; young: less than 5 years) as for other age groups (5 to 65 years). However, for below-poverty level nonwhite individuals, NO_2_ concentrations are notably higher for young children (3.0 ppb; 23%, *p*<0.01) and elderly people (3.1 ppb; 24%, *p*<0.01) than for the rest of the population (age 5 to 65 years, including whites and nonwhites).

An important issue is whether the NO_2_ disparities described above are relevant to public health. To investigate that question, we consider here one illustrative example: ischemic heart disease (IHD) annual deaths associated with NO_2_ concentration disparities between nonwhites and whites. Assuming a 6.6% change in IHD mortality rate per 4.1 ppb NO_2_ [39] and US-average IHD annual mortality rates (109 deaths per 100,000 people [40]), reducing NO_2_ concentrations to levels experienced by whites (a 4.6 ppb [38%] reduction) for all nonwhites (87 million people) would be associated with a decrease of ∼7,000 IHD deaths per year. For comparison, interventions with a similar benefit (a decrease in ∼7,000 IHD deaths per year) include: 16 million people increasing physical activity level from inactive (0 h/wk) to sufficiently active (>2.5 h/wk)[41]; 25 million people increasing physical activity level from insufficiently active (<2.5 h/wk) to sufficiently active (>2.5 h/wk); or, 3.2 million fewer adults (age 30–44) beginning smoking [42]. Calculations in this paragraph (details in **Table S2** in **File S1**) may underestimate true health impacts because we ignore here differences in vulnerability and susceptibility to air pollution and differences in underlying IHD mortality rates; also, the analysis above considers only one health outcome (IHD mortality) and one pollutant (outdoor NO_2_).

**Figure 1:**
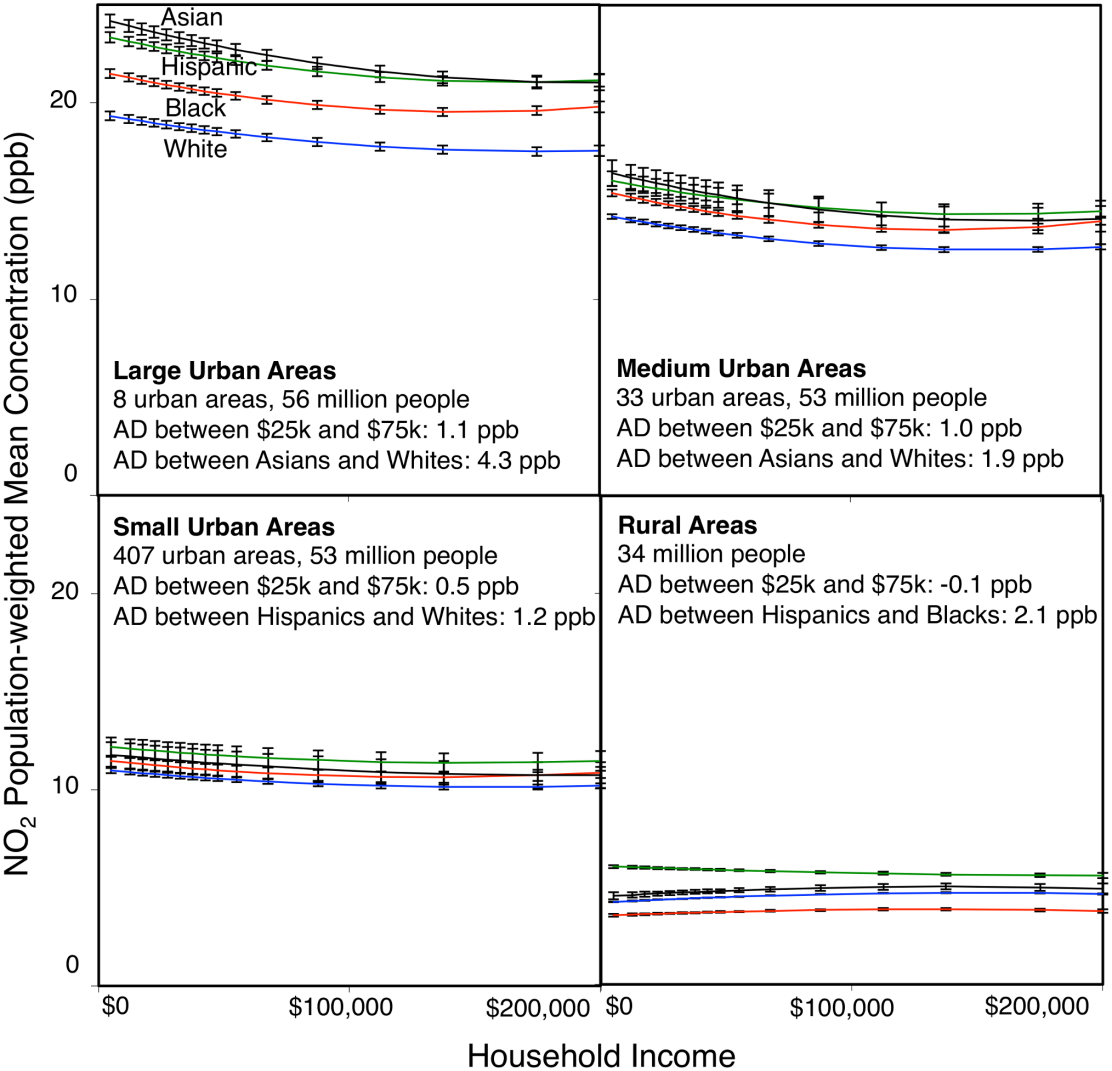
Within-urban and within-rural population-weighted mean NO_2_ concentrations (105 million householders) by Census household income category, race, and urban category (large UA population tertile, medium UA population tertile, small UA population tertile, or rural). Concentrations shown are modeled by UA population tertile (linear regressions: *R^2^*>0.98 [large UAs], >0.96 [medium UAs], >0.86 [small UAs], >0.47 [rural]; all models are statistically significant at *p*<0.01*;* see **Tables S3–S18** in **File S1**). For visual display, plots use the population-weighted mean UA-specific dummy variable for each UA population tertile. Error bars show the 95% confidence intervals on linear regression model predictions. AD = average difference, UA = Urban Area. AD values shown are for interquartile range incomes ($25k, $75k) and for race-ethnicity groups with highest and lowest concentrations for that panel.

Within individual urban areas, even after controlling for urban area size and household income group, nonwhites are generally more exposed to residential outdoor NO_2_ air pollution than whites. **Figure 1** presents regression models predicting population-weighted mean NO_2_ concentration as a function of household income for all 16 Census-defined household income categories and for the 4 largest race-ethnicity groups (Whites, Hispanics, Blacks, Asians) by urban area size (small; medium; large; defined by urban population tertiles). Each within-urban model reveals an inverse relationship between population-weighted NO_2_ concentration and household income with high statistical significance (*R^2^*>0.86; model *p*-value<0.01; **Tables S3–S18** in **File S1**). Across household income groups, urban NO_2_ concentrations are often highest for Asians or Hispanics and lowest for Whites.

Within individual urban areas, on average, NO_2_ concentration disparities by race (after controlling for income) are more than 2 times greater than NO_2_ concentration disparities by income (after controlling for race). The relative importance of race versus income for environmental injustice increases with urban area size. For each urban area size category, we compared average differences in NO_2_ concentrations between the race group (of the 4 largest race groups) with the highest versus the lowest NO_2_ concentrations (controlling for household income group) to the average differences in NO_2_ concentrations between the $25,000 versus $75,000 income groups (approximate income interquartile range; controlling for race group; **Figure 1**). In large urban areas, disparities by race are ∼4 times greater than by income. In medium and small urban areas, disparities by race are ∼2 times greater than by income. For rural residents, differences by race are ∼20 times greater than by income (despite significantly lower average concentrations for rural versus urban residents: 4.4 ppb [rural population-weighted mean] versus 14.2 ppb [urban population-weighted mean]). For rural areas, differences by income are small (0.1 ppb) and in the opposite direction as for the US as a whole (i.e., in rural areas, concentrations are higher for higher- than for lower-income groups).

As an alternative analysis, we developed NO_2_ regression models for which each observation is a Block Group concentration rather than population-weighted concentration (by location, income and race category; **Tables S19–S30** in **File S1**). Results for the Block Group and population-weighted analyses cannot be compared directly. Block Group analyses indicate a more varied relationship with race and with income, but in general suggest that NO_2_ concentrations are higher for nonwhites than for whites and are higher for lower-income than for higher-income communities; and, on average, disparities are greater by race (percent white) than by income.

Inequality metrics are presented in **Table 3**. On a national scale, we find that inequality levels are higher for NO_2_ (Atkinson Index = 0.11) than for income (Atkinson Index = 0.08), despite the fact that the US has a high degree of income inequality compared to most developed nations [43].

**Table 3:** Environmental injustice and inequality metric mean (population-weighted mean) [range].

	Environmental Injustice	Environmental Inequality
	Difference^1^ between low-income nonwhites and high-income whites (ppb)	Atkinson Index^2^
National	3.4	0.11
* Urban*	2.8	0.059
* Mixed*	0.4	0.062
* Rural*	−0.3	0.080
Regions (*n = *10)	3.6 (3.7) [1.1 to 7.1]	0.083 (0.083) [0.064 to 0.12]
States (*n* = 49)	2.5 (3.5) [−0.6 to 7.2]	0.068 (0.073) [0.006 to 0.14]
Counties^3^ (*n* = 3,109)	0.8 (1.9) [−2.6 to 7.0]	0.031 (0.027) [0.000006 to 0.17]
Urban Areas (*n* = 448)	1.3 (2.8) [−1.1 to 6.0]	0.009 (0.016) [0.00008 to 0.040]
* Large Urban Areas* (*n* = 8)	3.6 (4.0) [0.8 to 6.0]	0.018 (0.020) [0.009 to 0.031]
* Medium Urban Areas* (*n* = 33)	2.6 (2.7) [1.1 to 5.0]	0.015 (0.015) [0.005 to 0.039]
* Small Urban Areas* (*n* = 407)	1.1 (1.7) [−1.1 to 4.7]	0.009 (0.012) [0.0001 to 0.040]

^*1*^Larger positive differences indicate greater injustice (concentrations are higher for low-income nonwhites than for high-income whites). A negative value denotes concentrations being lower for low-income nonwhites than for high-income whites.

^*2*^Larger Atkinson Indices indicate greater inequality. Inequality aversion coefficient: ε = 0.75.

^*3*^This analysis excludes counties that consist of 1 Block Group (*n = *29; total population = 21,500 people) or contain 0 low-income nonwhites and/or 0 high-income whites (*n* = 16; total population = 65,800 people).

**Figure 2:**
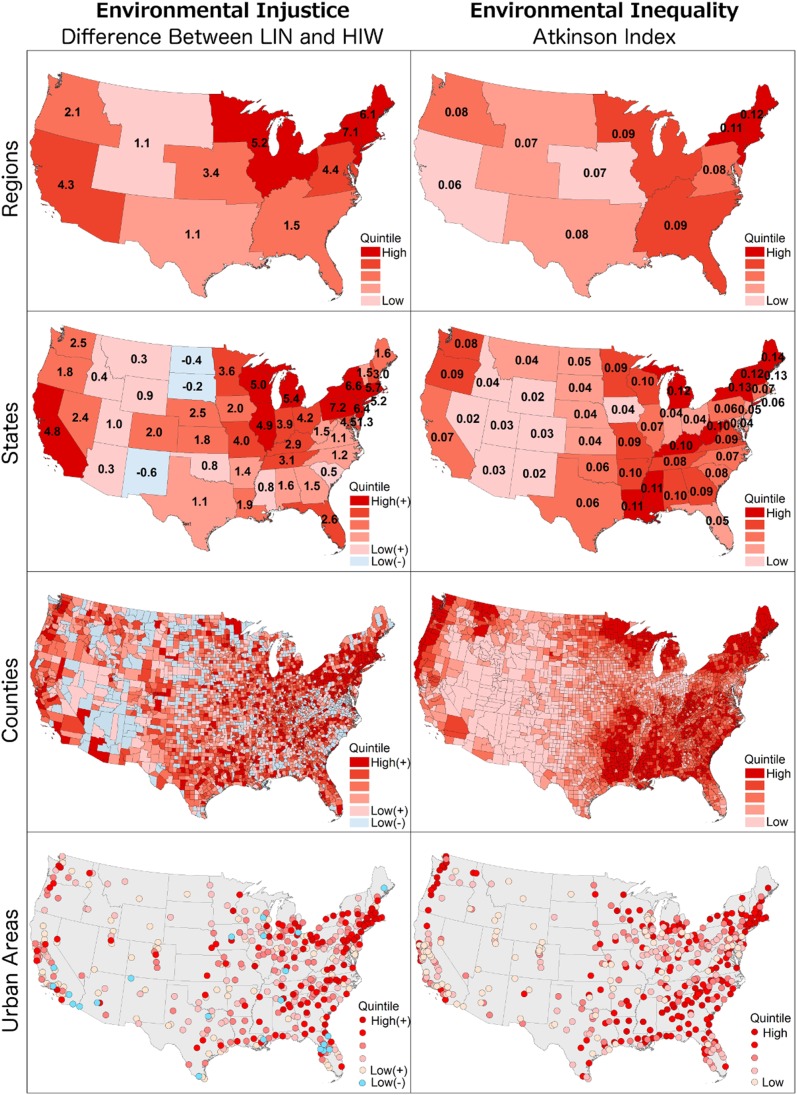
Environmental injustice and inequality in residential outdoor NO_2_ concentrations for US regions, states, counties and urban areas. The left column shows differences in population-weighted mean NO_2_ concentrations between low-income nonwhites (LIN) and high-income whites (HIW), with larger positive differences (red colors) indicating higher injustice (larger concentration difference between LIN and HIW). The right column shows the Atkinson Index, with higher values indicating greater inequality.

**Figure 2** **.** shows national spatial patterns in environmental injustice and inequality in outdoor NO_2_ air pollution. States with high levels (top quintile) of both injustice and inequality include New York, Michigan, and Wisconsin. Given previous work documenting inequality and injustice in NO_2_ concentrations (among other environmental hazards) it is not surprising that we observe injustice and inequality in NO_2_ concentrations on a national basis. What is unexpected, however, are the spatial patterns in **Figure 2**. Environmental injustice and inequality do not exhibit clear spatial coherence with respect to regional race or income characteristics. For example, among urban areas, environmental inequality (Atkinson Index) has a low correlation with race (percent nonwhite) and average income [Pearson’s *r*<0.2]. Understanding the processes driving these spatial distributions of environmental injustice and inequality is thus a priority need for future research.

Inequality and injustice metrics vary by location. NO_2_ inequality (Atkinson Index) is slightly higher among rural residents than among urban residents, but environmental injustice may be higher for urban residents: NO_2_ concentration differences between lower-income nonwhites and higher-income whites are an order of magnitude higher and in the opposite direction for urban residents as for rural residents (2.8 ppb versus −0.3 ppb; see **Table 1**). Across the 448 urban areas in the US, there is variation in injustice (difference range [ppb]: −1.1 to 6.0) and inequality (Atkinson Index range: 0.00008 to 0.04) for NO_2_ air pollution, consistent with a previous multi-city study [13]. In 426 of 448 urban areas (accounting for 99% of the total US urban population), NO_2_ concentrations are higher for the lower-income nonwhite group than for the higher-income white group, with injustice and inequality tending to be higher in large urban areas. Supporting Information (**File S2**) provides environmental injustice and inequality rankings by urban area, county, and state.

A contribution of this work is that it covers the entire contiguous US population, including both urban and rural populations, with higher spatial precision in urban areas (urban BG-scale: ∼1-km; LUR scale: ∼0.1-km) relative to previous regional or multi-city air quality environmental equality and/or justice studies (typical air quality model-scale: ∼12-km grid or coarser). Although the spatial resolution is higher than in previous work, resolution is still a limitation: because we are using Census demographic data, we are unable to study within-BG variations. As a second limitation, we measure inequality for one pollutant (NO_2_); inequality may differ for other pollutants (e.g., ozone [44]) or for multi-pollutant cumulative exposure [32]. As a third limitation, we study only ambient pollution; disparities may also exist for indoor NO_2_ emissions (e.g., owing to indoor sources such as natural gas combustion), for indoor-outdoor pollution relationships (e.g., because low-income households may live in comparatively older, leakier buildings), and for occupational and commute exposures. As a fourth limitation, there is a temporal mismatch between the year-2000 Census data and year-2006 air pollution data. We expect demographic changes during that time to be small compared to the cross-sectional differences explored here.

We investigated environmental injustice and inequality in residential outdoor NO_2_ air pollution for the contiguous US population. Nationally, inequality in average NO_2_ concentration is greater than inequality in average income. Nonwhites experience 4.6 ppb (38%) higher residential outdoor NO_2_ concentrations than whites – an exposure gap that has potentially large impacts to public health. Within individual urban areas, after controlling for income, nonwhites are on average exposed to higher outdoor residential NO_2_ concentrations than whites; and, after controlling for race, lower-income populations are exposed to higher outdoor residential average NO_2_ concentrations than higher-income populations. The spatial patterns observed for inequality and injustice nationally (**Figure 2**) are not predicted by region, race, or income. Our results highlight a need for future work exploring the reasons behind these spatial distributions of environmental injustice and inequality. Results given here provide strong US-wide evidence of ambient NO_2_ air pollution injustice and inequality, establish a national context for studies of individual metropolitan areas and regions, and enable comprehensive tracking over time. Hopefully results given here will usefully allow policy-makers to identify counties and urban areas with highest priority NO_2_ air pollution environmental justice and equality concerns.

## Supporting Information

File S1 (PDF)

File S2 (XLSX)
