# Supplementary material for: National Patterns in Environmental Injustice and Inequality: Outdoor NO2 Air Pollution in the United States
Source: PLoS One. 2014 Apr 15;9(4):e94431. doi: 10.1371/journal.pone.0094431 (PMC3988057; doi:10.1371/journal.pone.0094431)
Supplement: File S1 — (PDF) [file pone.0094431.s002.pdf]

Supporting Information

Title: National patterns in environmental injustice and inequality: outdoor NO_2 air pollution in the United States
Authors: Lara P. Clark, Dylan B. Millet, Julian D. Marshall

Filse S1 Contents:
42 pages (.pdf)
Figures S1-S2
Tables S1-S30

File S2 Contents:
Spreadsheet of environmental injustice and inequality rankings for states, counties, and urban areas (.xls)

Supporting information for environmental injustice and inequality metrics

Equation S1 presents the calculation of population-weighted NO_2 concentration (C), where i indexes the Block Groups, c_i is the mean concentration for each Block Group i; p_i is the population of Block Group i; and n is the number of Block Groups. As an example, for calculating the population-weighted NO_2 concentration for urban whites, c_i is the mean concentration for each urban Block Group i; p_i is the white population of urban Block Group i; and n is the number of urban Block Groups

(Equation S1)
$$C = \frac{\sum_{i=1}^{n} c_i p_i}{\sum_{i=1}^{n} p_i}$$

Equations S2-S3 present the calculation of the Atkinson Index (A) for grouped Census data [1,2], under two conditions for the inequality aversion parameter (ε): ε = 1 (**Equation S2**) or ε ≠ 1 (**Equation S3**). Here, i indexes the Block Groups within the geographical unit of interest (e.g., a specific state, county, or urban area), c is the mean concentration in Block Group i; f_i is the fraction of total population of the geographical unit of interest in Block Group i; c_i is the mean concentration in Block Group i; and w is the population-weighted mean concentration among Block Groups in the geographical unit of interest.

(Equation S2)
$$A = 1 - \exp\left(\left[\frac{1}{n}\right]\sum_{i=1}^{n} f_i \log\left(\frac{c_i}{w}\right)\right)$$

(Equation S3) $$A = 1 - \left(\left[\frac{1}{n} \right] \sum_{i=1}^{n} f_i \left(\frac{c_i}{w} \right)^{(1-\varepsilon)} \right)^{\left(\frac{1}{1-\varepsilon} \right)}$$

Figure S1 presents a sensitivity analysis on the selection of the Atkinson Index (with inequality aversion parameter, $\varepsilon = 0.75$) as the core environmental inequality metric presented in the main text. This core environmental inequality metric is highly correlated (Pearson's correlation coefficients > |0.96| and Spearman's rank coefficients > |0.98|) with the alternate environmental inequality metrics we considered (Atkinson Indices with $\varepsilon = \{0.25, 0.5, 1, 1.25, 1.5, 2\}$, Gini coefficient, and Gini coefficents on modified and inverse NO_2 datasets) among the 448 urban areas. Thus, the conclusions presented in the main text are not highly sensitive to the core metric selection for environmental inequality.

As a supplement to **Figure 2** and **Table 3** in the main text, **Figure S2** and **Table S1** present alternate metrics for environmental injustice (relative percent difference between lower-income nonwhites and higher-income whites) and inequality (Gini coefficient) for US regions, states, counties and urban areas.

Supporting information for health impact estimates

Table S2 provides details for the public health impacts (reductions in Ischemic Heart Disease mortality) associated with disparities in NO_2 concentration differences observed between nonwhites and whites.

Supporting information for regression models

Tables S3-S18 present linear regression model details for **Figure 1** in the main text. The dependent variable in each model is the population-weighted NO_2 concentration for Census householders. The independent variables are income, income-squared, and, for urban models, a dummy variable to control for specific urban area. We developed separate regression models for each of the 4 largest race-ethnicity categories (white, black, hispanic, asian) in 4 location categories (large urban areas, medium urban areas, small urban areas, rural areas), yielding 16 total regression models.

As an alternative analysis to **Figure 1** in the main text, **Tables S19-S30** present NO_2 regression models for which each observation is a Block Group concentration rather than population-weighted concentration. The dependent variable for each model is the Block Group mean NO_2 concentration. The independent variables are Block Group average income, Block Group average income-squared, and Block Group percent white population. We developed separate regression models for each of the 3 Block Group percent white population tertiles and for each of 4 location categories (large urban areas, medium urban areas, small urban areas, and rural areas), yielding 12 total regression models. Compared to the population-weighted concentration analyses (**Figure 1**; **Tables S3-S18**), Block Group analyses indicate a more varied relationship with race and with income, but in general suggest that NO_2 concentration disparities are greater by race (percent white tertile) than by income.

Supporting information references

1. Laporte A (2002) A note on the use of a single inequality index in testing the effect of income distribution on mortality. Soc Sci Med 55: 1561-1570.
2. Volscho TW, Fullerton AS (2005) Metropolitan earnings inequality: union and government-sector employment effects. Soc Sci Quart S86: 1326-1337.
3. Jerrett M, Burnett RT, Beckerman BS, Turner MC, Krewski D, et al. (2013) Spatial analysis of air pollution and mortality in California. Am J Resp Crit Care 188: 593-599
4. World Health Organization. Comparative Quantification of Health Risks: Global and Regional Burden of Disease Attribution to Selected Major Risk Factors. Available: http://www.who.int/publications/cra/chapters/volume1/0729-0882.pdf. Accessed: 1 October 2013.
5. Danaei G, Ding EL, Mozaffarian D, Taylor B, Rehm J, et al. (2009) The preventable causes of death in the United States: comparative risk assessment of dietary, lifestyle, and metabolic risk factors. PLOS Med 6: 10.1371/journal.pmed.1000058.
6. U. S. Census Bureau. 2000 Decennial Census. Available: https://www.census.gov/main/www/cen2000.html. Accessed: 1 April 2012.
7. U. S. Centers for Disease Control. National Vital Statistics Reports: Deaths, Preliminary Data for 2011. Available: http://www.cdc.gov/nchs/data/nvsr/nvsr61/nvsr61_06.pdf. Accessed: 1 October 2013.
8. U. S. Centers for Disease Control. Smoking and tobacco use fast facts, 2011. Available: http://www.cdc.gov/tobacco/data_statistics/fact_sheets/fast_facts/. Accessed: 1 March 2014.

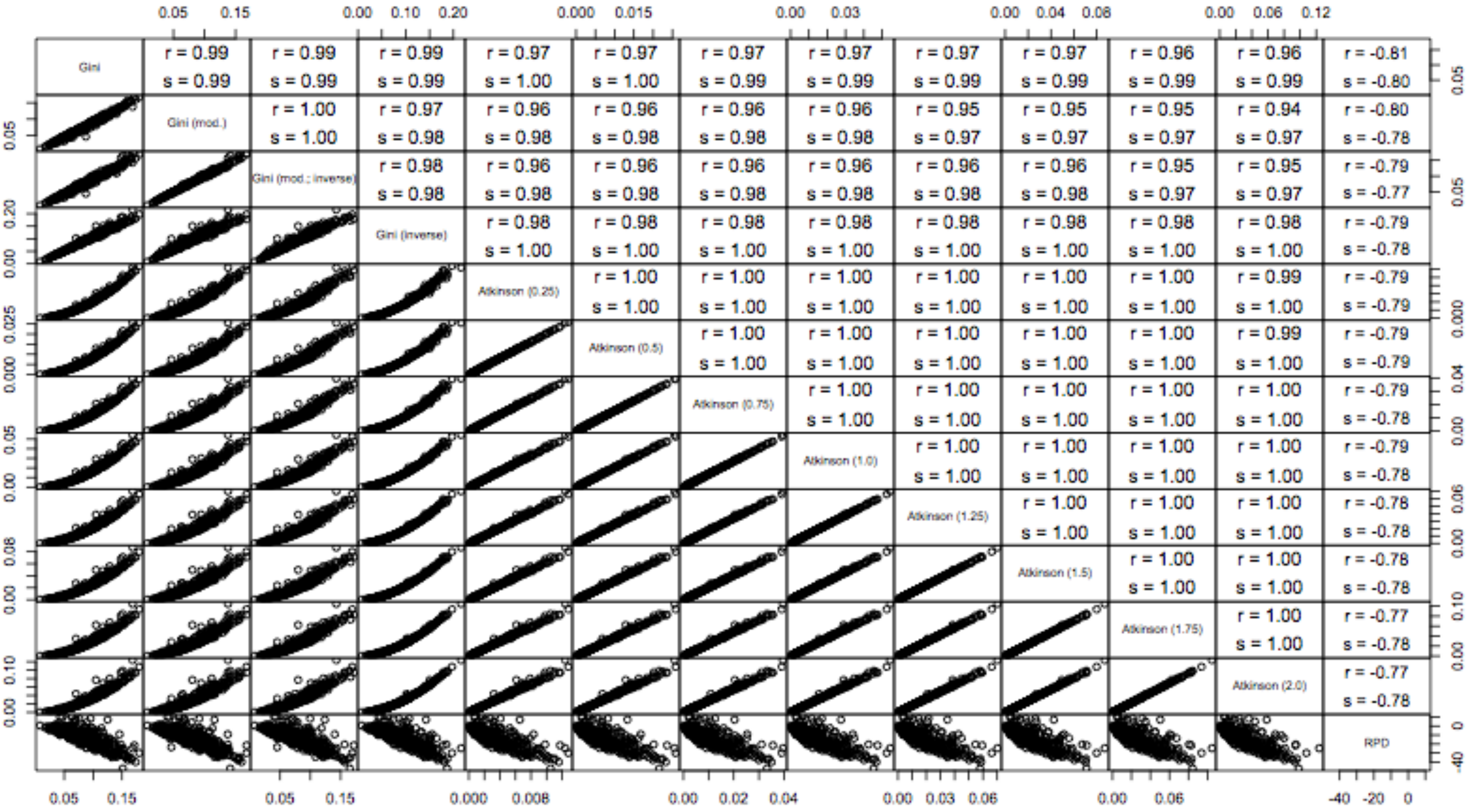

Figure S1. Correlations among environmental injustice and inequality metrics (Pearson's correlation coefficient, r; Spearman's rank correlation coefficient, s) for urban areas (*n*=448). "Atkinson (0.75)" indicates Atkinson Index calculated with the inequality aversion parameter (ε) = 0.75. "Gini (mod.)" indicates the Gini Coefficient calculated on a modified NO_2 dataset in which the BGs with the lowest 10% of NO_2 concentrations in each UA are clipped to the 10th percentile concentration in the UA. "Gini (inverse)" indicates the Gini Coefficient calculated using the inverse of concentration (ppb^{-1}) for all BGs.

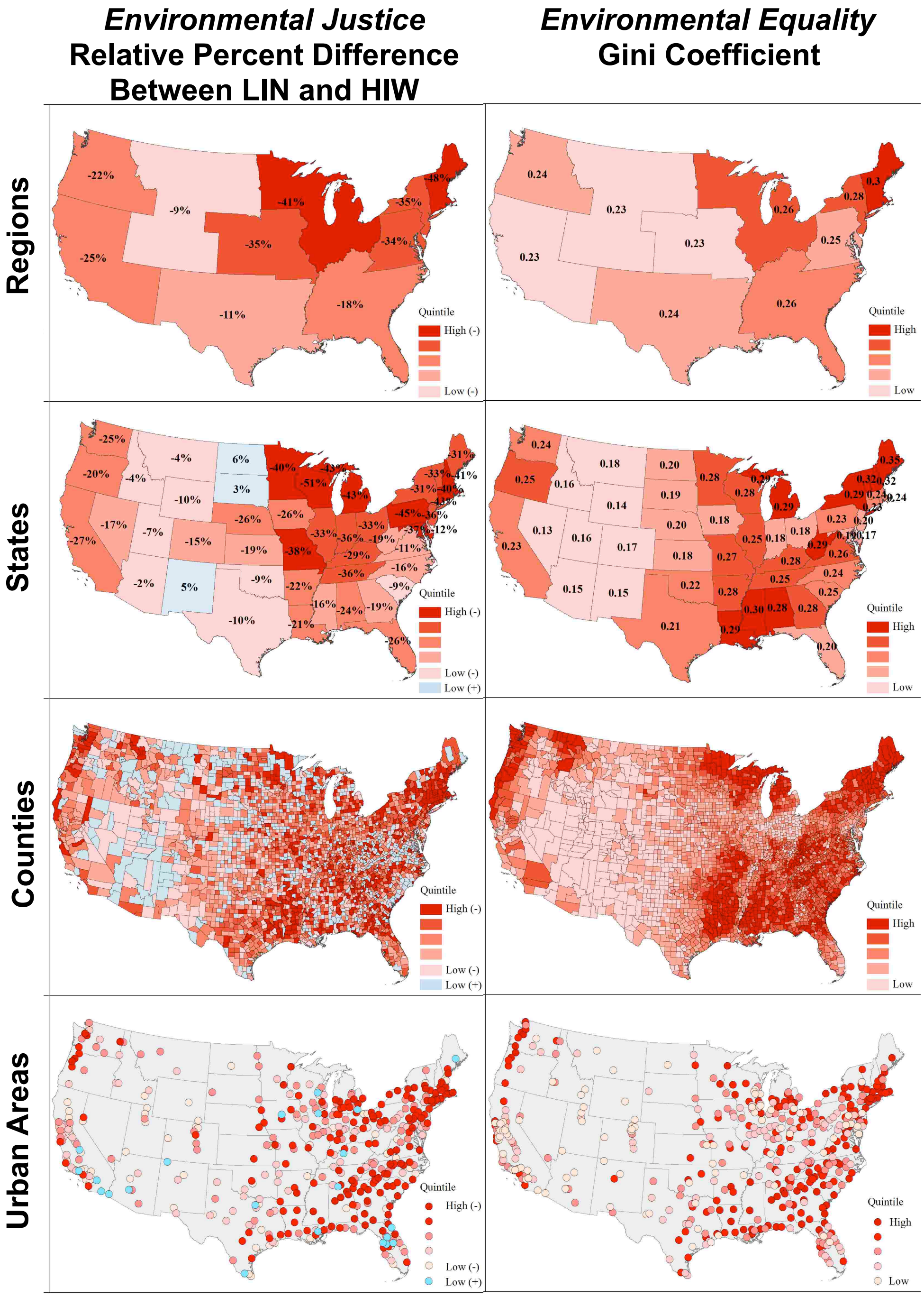

Figure S2. Supplemental environmental injustice and inequality in residential outdoor NO_2 concentrations for US regions, states, counties and urban areas. The left column shows relative difference in population-weighted mean NO_2 concentration between low-income nonwhites and high-income whites, with larger positive differences (red colors) indicating higher injustice (larger relative percent difference between lower-income nonwhites and higher-income whites). The right column shows the Gini Coefficient, with higher values indicating greater inequality.

Table S1. Supplemental environmental injustice and inequality metric means (ranges)

	Environmental Injustice<br>Difference[2] in population-weighted concentration between low-income nonwhites and high-income whites (%)	Environmental Inequality<br>Gini Coefficient[1]
National	27%	0.30
Urban	19%	0.23
Mixed	5%	0.22
Rural	-7%	0.26
Regions (*n* =10)	28% (9% to 48%)	0.25 (0.23 to 0.30)
States (*n* = 49)	23% (6% to 51%)	0.23 (0.08 to 0.35)
Counties[3] (*n* = 3,109)	11% (-52% to 67%)	0.14 (0.0008 to 0.38)
Urban Areas (*n* = 448)	12% (11% to 47%)	0.08 (0.008 to 0.18)

[1]Larger Gini Coefficients indicate greater inequality.
[2]Larger positive percent differences indicate greater injustice (low-income nonwhites more exposed relative to high-income whites). Negative differences indicate that high-income whites are more exposed relative to low-income nonwhites.
[3]This analysis excludes counties that consist of 1 Block Group (*n*=29; total population = 21,500 people) or contain 0 low-income nonwhites and/or 0 high-income whites (*n*=16; total population = 65,800 people).

Table S2. Public health impact data and calculations

Data for calculations	Value	Source
NO_2 population-weighted concentrations		
Nonwhites	14.5 ppb	Table 1
Whites	9.9 ppb	Table 1
Difference	4.6 ppb	Table 1
Relative risks in Ischemic Heart Disease mortality		
Increasing NO_2 concentrations by 4.1 ppb	1.066	Jerrett et al., 2013 [3]
NO_2 concentrations experienced by nonwhites (14.5 ppb)	1.254[a]	Table S2[a]
NO_2 concentrations experienced by whites (9.9 ppb)	1.167[a]	Table S2[a]
Increasing physical activity level from inactive (0 h/wk) to sufficiently active (>2.5 h/wk)	1.47[b]	WHO 2004 [4]
Increasing physical activity level from insufficient (<2.5 h/wk) to sufficiently active (>2.5 h/wk)	1.31[b]	WHO 2004 [4]
Nonsmoking versus smoking status (adults age 30-44 years)	3.9[c]	Danaei et al., 2009 [5]
Population data		
Nonwhite population	87 million	Census 2000 [6]
Ischemic Heart Disease mortality rate	109 deaths per 100,000 people	CDC 2013 [7]

[a]Relative risks (RR) for NO_2 concentrations experienced by nonwhites and whites calculated using: RR = $exp(\beta c)$, where c is the NO_2 concentration (units: ppb), and $\beta = ln(1.066)/(4.1 \text{ ppb}) = 0.0156 \text{ ppb}^{-1}$.

[b]Since ~29% of the US adult population is physically inactive, ~45% is insufficiently physically active, and ~26% is sufficiently physically active [4], based on an overall IHD annual mortality of 109 (units: deaths per 100,000 people), IHD annual mortality would be 125.6 for physically inactive adults, 111.9 for insufficiently active adults, and 85.4 for sufficiently active adults. Thus, the annual risk difference attributable to increasing physical activity level from inactive to sufficiently active is 125.6 - 85.4 = **40.2 IHD deaths per 100,000 people**; and, the annual risk difference attributable to increasing physical activity level from insufficiently to sufficiently active is 111.9 - 85.4 = **26.5 IHD deaths per 100,000 people**.

[c]Relative risk (RR) for IHD mortality for smoking versus non-smoking adults age 30-44 years: 5.5 (men); 2.3 (women). Thus, the average RR (for both men and women) is 3.9. Since ~18% of the US adult population smokes [8], based on an overall IHD annual mortality of 109 (units: deaths per 100,000 people), IHD annual mortality would be 279.3 for smokers, 71.6 for nonsmokers; the annual risk difference attributable to changing smoking status is 279.3 – 71.6 = **207.7 IHD deaths per 100,000 people**.

Ischemic Heart Disease (IHD) mortality reduction per year associated with reducing annual NO_2 concentrations for all nonwhites to levels experienced by whites:

$$87{,}000{,}000 \text{ people} \times \frac{109 \text{ IHD deaths}}{100{,}000 \text{ people}} \times \frac{1.254 - 1.0}{1.254} = 19{,}208 \text{ IHD deaths}$$

$$87{,}000{,}000 \text{ people} \times \frac{109 \text{ IHD deaths}}{100{,}000 \text{ people}} \times \frac{1.167 - 1.0}{1.254} = 12{,}629 \text{ IHD deaths}$$

Difference =19,208 - 12,629 = **6,579 IHD deaths per year**

Number of people changing from smoking to nonsmoking status associated with a reduction of 6,579 IHD deaths per year:

$$6{,}579 \text{ IHD deaths} \times \frac{100{,}000 \text{ people}}{207.7 \text{ IHD deaths}} = \mathbf{3.2 \text{ million people}}$$

Number of people changing physical activity status from inactive to sufficiently active associated with a reduction of 6,579 IHD deaths per year:

$$6{,}579 \text{ IHD deaths} \times \frac{100{,}000 \text{ people}}{40.2 \text{ IHD deaths}} = \mathbf{16 \text{ million people}}$$

Number of people changing physical activity status from insufficiently active to sufficiently active associated with a reduction of 6,579 IHD deaths per year:

$$6{,}579 \text{ IHD deaths} \times \frac{100{,}000 \text{ people}}{26.5 \text{ IHD deaths}} = \mathbf{25 \text{ million people}}$$

Table S3. Linear regression model results for population-weighted concentrations for White householders in large Urban Areas

Variable	Coefficient	*p*-value
(Intercept)	15.62	0.0000***
Income[a]	-1.35E-05	0.0000***
Income[a]-squared	6.36E-11	0.0000***
Urban Area-specific dummy variables[b]		
Dallas--Fort Worth--Arlington, TX Urbanized Area	-2.24	0.0000***
Detroit, MI Urbanized Area	-1.00	0.0000***
Los Angeles--Long Beach--Santa Ana, CA Urbanized Area	7.36	0.0000***
Miami, FL Urbanized Area	-4.11	0.0000***
New York--Newark, NY--NJ--CT Urbanized Area	5.98	0.0000***
Philadelphia, PA--NJ--DE--MD Urbanized Area	0.48	0.0004***
Washington, DC--VA--MD Urbanized Area	-2.50	0.0000***
Model adjusted $R^2 = 0.98$		
Model *p*-value = 0.0000***		
n = 128		

[a]Income is the mid-point of the Census household income category, transformed by subtracting the mean household income.
[b]The reference UA (for which the UA specific dummy variable = 0) is Chicago, IL.

Statistical significance: *p<0.1; **p<0.05; ***p<0.01

Table S4. Linear regression model results for population-weighted concentrations for Black householders in large Urban Areas

Variable	Coefficient	*p*-value
(Intercept)	17.46	0.0000***
Income[a]	-1.54E-05	0.0000***
Income[a]-squared	9.60E-11	0.0000***
Urban Area-specific dummy variables[b]		
Dallas--Fort Worth--Arlington, TX Urbanized Area	-4.35	0.0000***
Detroit, MI Urbanized Area	0.10	0.47
Los Angeles--Long Beach--Santa Ana, CA Urbanized Area	8.18	0.0000***
Miami, FL Urbanized Area	-4.60	0.0000***
New York--Newark, NY--NJ--CT Urbanized Area	7.59	0.0000***
Philadelphia, PA--NJ--DE--MD Urbanized Area	1.53	0.0000***
Washington, DC--VA--MD Urbanized Area	-3.67	0.0000***

Model adjusted R^2 = 0.99

Model *p*-value = 0.0000***

n = 128

[a]Income is the mid-point of the Census household income category, transformed by subtracting the mean household income.
[b]The reference UA (for which the UA specific dummy variable = 0) is Chicago, IL.

Statistical significance: *p<0.1; **p<0.05; ***p<0.01

Table S5. Linear regression model results for population-weighted concentrations for Hispanic householders in large Urban Areas

Variable	Coefficient	*p*-value
(Intercept)	17.85	0.0000***
Income[a]	-1.73E-05	0.0000***
Income[a]-squared	8.71E-11	0.0000***
Urban Area-specific dummy variables[b]		
Dallas--Fort Worth--Arlington, TX Urbanized Area	-4.09	0.0000***
Detroit, MI Urbanized Area	-2.22	0.0000***
Los Angeles--Long Beach--Santa Ana, CA Urbanized Area	7.98	0.0000***
Miami, FL Urbanized Area	-5.22	0.0000***
New York--Newark, NY--NJ--CT Urbanized Area	6.98	0.0000***
Philadelphia, PA--NJ--DE--MD Urbanized Area	0.87	0.0000***
Washington, DC--VA--MD Urbanized Area	-4.00	0.0000***

Model adjusted $R^2 = 0.99$

Model *p*-value = 0.0000***

n = 128

[a]Income is the mid-point of the Census household income category, transformed by subtracting the mean household income.
[b]The reference UA (for which the UA specific dummy variable = 0) is Chicago, IL.

Statistical significance: *$p<0.1$; **$p<0.05$; ***$p<0.01$

Table S6. Linear regression model results for population-weighted concentrations for Asian householders in large Urban Areas

Variable	Coefficient	*p*-value
(Intercept)	16.78	0.0000***
Income[a]	-2.23E-05	0.0000***
Income[a]-squared	8.94E-11	0.0000***
Urban Area-specific dummy variables[b]		
Dallas--Fort Worth--Arlington, TX Urbanized Area	-3.06	0.0000***
Detroit, MI Urbanized Area	-1.78	0.0000***
Los Angeles--Long Beach--Santa Ana, CA Urbanized Area	8.09	0.0000***
Miami, FL Urbanized Area	-4.94	0.0000***
New York--Newark, NY--NJ--CT Urbanized Area	7.91	0.0000***
Philadelphia, PA--NJ--DE--MD Urbanized Area	0.82	0.0000***
Washington, DC--VA--MD Urbanized Area	-3.64	0.0000***

Model adjusted R^2 = 0.99

Model *p*-value = 0.0000***

n = 128

[a]Income is the mid-point of the Census household income category, transformed by subtracting the mean household income.
[b]The reference UA (for which the UA specific dummy variable = 0) is Chicago, IL.

Statistical significance: *$p<0.1$; **$p<0.05$; ***$p<0.01$

Table S7. Linear regression model results for population-weighted concentrations for White householders in medium Urban Areas

Variable	Coefficient	*p*-value
(Intercept)	10.51	0.0000***
Income[a]	-1.33E-05	0.0000***
Income[a]-squared	7.70E-11	0.0000***
Urban Area-specific dummy variables[b]		
Austin, TX Urbanized Area	0.54	0.0000***
Baltimore, MD Urbanized Area	2.58	0.0000***
Boston, MA--NH--RI Urbanized Area	4.05	0.0000***
Buffalo, NY Urbanized Area	1.15	0.0000***
Cincinnati, OH--KY--IN Urbanized Area	1.80	0.0000***
Cleveland, OH Urbanized Area	3.56	0.0000***
Columbus, OH Urbanized Area	2.86	0.0000***
Denver--Aurora, CO Urbanized Area	5.82	0.0000***
Houston, TX Urbanized Area	2.70	0.0000***
Indianapolis, IN Urbanized Area	1.87	0.0000***
Kansas City, MO--KS Urbanized Area	0.80	0.0000***
Las Vegas, NV Urbanized Area	4.61	0.0000***
Louisville, KY--IN Urbanized Area	1.94	0.0000***
Memphis, TN--MS--AR Urbanized Area	-0.36	0.0000***
Milwaukee, WI Urbanized Area	0.88	0.0000***
Minneapolis--St. Paul, MN Urbanized Area	-0.46	0.0000***
New Orleans, LA Urbanized Area	2.52	0.0000***
Orlando, FL Urbanized Area	-0.25	0.0047***
Phoenix--Mesa, AZ Urbanized Area	3.75	0.0000***
Pittsburgh, PA Urbanized Area	5.02	0.0000***
Portland, OR--WA Urbanized Area	0.77	0.0000***
Providence, RI--MA Urbanized Area	2.32	0.0000***
Riverside--San Bernardino, CA Urbanized Area	8.55	0.0000***
Sacramento, CA Urbanized Area	3.33	0.0000***

St. Louis, MO--IL Urbanized Area	0.59	0.0000***
San Antonio, TX Urbanized Area	1.34	0.0000***
San Diego, CA Urbanized Area	3.58	0.0000***
San Francisco--Oakland, CA Urbanized Area	5.47	0.0000***
San Jose, CA Urbanized Area	7.42	0.0000***
Seattle, WA Urbanized Area	1.01	0.0000***
Tampa--St. Petersburg, FL Urbanized Area	0.05	0.5599
Virginia Beach, VA Urbanized Area	0.39	0.0000***
Model adjusted R^2 = 0.99		
Model *p*-value = 0.0000***		
n = 528		

[a]Income is the mid-point of the Census household income category, transformed by subtracting the mean household income.
[b]The reference UA (for which the UA specific dummy variable = 0) is Atlanta, GA.

Statistical significance: **p*<0.1; ***p*<0.05; ****p*<0.01

Table S8. **Linear regression model results for population-weighted concentrations for Black householders in medium Urban Areas**

Variable	Coefficient	*p*-value
(Intercept)	11.47	0.0000***
Income[a]	-1.50E-05	0.0000***
Income[a]-squared	1.08E-10	0.0000***
Urban Area-specific dummy variables[b]		
Austin, TX Urbanized Area	-0.30	0.0080**
Baltimore, MD Urbanized Area	3.23	0.0000***
Boston, MA--NH--RI Urbanized Area	6.05	0.0000***
Buffalo, NY Urbanized Area	2.50	0.0000***
Cincinnati, OH--KY--IN Urbanized Area	2.54	0.0000***
Cleveland, OH Urbanized Area	4.60	0.0000***
Columbus, OH Urbanized Area	3.22	0.0000***
Denver--Aurora, CO Urbanized Area	5.17	0.0000***
Houston, TX Urbanized Area	2.30	0.0000***
Indianapolis, IN Urbanized Area	1.98	0.0000***
Kansas City, MO--KS Urbanized Area	0.80	0.0000***
Las Vegas, NV Urbanized Area	3.77	0.0000***
Louisville, KY--IN Urbanized Area	2.19	0.0000***
Memphis, TN--MS--AR Urbanized Area	-0.68	0.0000***
Milwaukee, WI Urbanized Area	2.26	0.0000***
Minneapolis--St. Paul, MN Urbanized Area	0.08	0.4947
New Orleans, LA Urbanized Area	1.97	0.0000***
Orlando, FL Urbanized Area	-0.69	0.0047****
Phoenix--Mesa, AZ Urbanized Area	3.09	0.0000***
Pittsburgh, PA Urbanized Area	6.49	0.0000***
Portland, OR--WA Urbanized Area	1.60	0.0000***
Providence, RI--MA Urbanized Area	3.92	0.0000***
Riverside--San Bernardino, CA Urbanized Area	8.27	0.0000***
Sacramento, CA Urbanized Area	2.80	0.0000***

St. Louis, MO--IL Urbanized Area	1.24	0.0000***
San Antonio, TX Urbanized Area	0.22	0.0574*
San Diego, CA Urbanized Area	4.06	0.0000***
San Francisco--Oakland, CA Urbanized Area	5.50	0.0000***
San Jose, CA Urbanized Area	6.67	0.0000***
Seattle, WA Urbanized Area	1.57	0.0000***
Tampa--St. Petersburg, FL Urbanized Area	-0.10	0.3770
Virginia Beach, VA Urbanized Area	0.73	0.0000***

Model adjusted $R^2 = 0.98$

Model *p*-value = 0.0000***

n = 528

[a]Income is the mid-point of the Census household income category, transformed by subtracting the mean household income.
[b]The reference UA (for which the UA specific dummy variable = 0) is Atlanta, GA.

Statistical significance: *p<0.1; **p<0.05; ***p<0.01

Table S9. Linear regression model results for population-weighted concentrations for Hispanic householders in medium Urban Areas

Variable	Coefficient	*p*-value
(Intercept)	11.66	0.0000***
Income[a]	-1.33E-05	0.0000***
Income[a]-squared	7.71E-11	0.0000***
Urban Area-specific dummy variables[b]		
Austin, TX Urbanized Area	-0.38	0.0042**
Baltimore, MD Urbanized Area	1.48	0.0000***
Boston, MA--NH--RI Urbanized Area	5.89	0.0000***
Buffalo, NY Urbanized Area	1.00	0.0000***
Cincinnati, OH--KY--IN Urbanized Area	1.35	0.0000***
Cleveland, OH Urbanized Area	4.71	0.0000***
Columbus, OH Urbanized Area	2.08	0.0000***
Denver--Aurora, CO Urbanized Area	5.68	0.0000***
Houston, TX Urbanized Area	2.70	0.0000***
Indianapolis, IN Urbanized Area	1.57	0.0000***
Kansas City, MO--KS Urbanized Area	1.09	0.0000***
Las Vegas, NV Urbanized Area	4.10	0.0000***
Louisville, KY--IN Urbanized Area	1.08	0.0000***
Memphis, TN--MS--AR Urbanized Area	-0.54	0.0000***
Milwaukee, WI Urbanized Area	1.50	0.0000***
Minneapolis--St. Paul, MN Urbanized Area	-0.27	0.0383**
New Orleans, LA Urbanized Area	1.34	0.0000***
Orlando, FL Urbanized Area	-1.13	0.0047****
Phoenix--Mesa, AZ Urbanized Area	3.44	0.0000***
Pittsburgh, PA Urbanized Area	5.00	0.0000***
Portland, OR--WA Urbanized Area	-0.42	0.0000***
Providence, RI--MA Urbanized Area	4.34	0.0000***
Riverside--San Bernardino, CA Urbanized Area	8.59	0.0000***
Sacramento, CA Urbanized Area	2.72	0.0000***

St. Louis, MO--IL Urbanized Area	0.12	0.3674
San Antonio, TX Urbanized Area	0.98	0.0000***
San Diego, CA Urbanized Area	3.09	0.0000***
San Francisco--Oakland, CA Urbanized Area	4.91	0.0000***
San Jose, CA Urbanized Area	6.83	0.0000***
Seattle, WA Urbanized Area	0.64	0.0000***
Tampa--St. Petersburg, FL Urbanized Area	-0.55	0.0000***
Virginia Beach, VA Urbanized Area	-0.50	0.0000***
Model adjusted R^2 = 0.98		
Model *p*-value = 0.0000***		
n = 528		

[a]Income is the mid-point of the Census household income category, transformed by subtracting the mean household income.
[b]The reference UA (for which the UA specific dummy variable = 0) is Atlanta, GA.

Statistical significance: *$p<0.1$; **$p<0.05$; ***$p<0.01$

Table S10. Linear regression model results for population-weighted concentrations for Asian householders in medium Urban Areas

Variable	Coefficient	*p*-value
(Intercept)	11.26	0.0000***
Income[a]	-1.84E-05	0.0000***
Income[a]-squared	9.34E-11	0.0000***
Urban Area-specific dummy variables[b]		
Austin, TX Urbanized Area	0.09	0.6063
Baltimore, MD Urbanized Area	1.62	0.0000***
Boston, MA--NH--RI Urbanized Area	5.97	0.0000***
Buffalo, NY Urbanized Area	0.88	0.0000***
Cincinnati, OH--KY--IN Urbanized Area	1.69	0.0000***
Cleveland, OH Urbanized Area	3.51	0.0000***
Columbus, OH Urbanized Area	2.21	0.0000***
Denver--Aurora, CO Urbanized Area	4.97	0.0000***
Houston, TX Urbanized Area	2.44	0.0000***
Indianapolis, IN Urbanized Area	1.20	0.0000***
Kansas City, MO--KS Urbanized Area	0.75	0.0000***
Las Vegas, NV Urbanized Area	4.19	0.0000***
Louisville, KY--IN Urbanized Area	1.60	0.0000***
Memphis, TN--MS--AR Urbanized Area	-0.41	0.0176**
Milwaukee, WI Urbanized Area	1.11	0.0000***
Minneapolis--St. Paul, MN Urbanized Area	-0.30	0.0858*
New Orleans, LA Urbanized Area	1.07	0.0000***
Orlando, FL Urbanized Area	-0.98	0.0047****
Phoenix--Mesa, AZ Urbanized Area	3.30	0.0000***
Pittsburgh, PA Urbanized Area	5.64	0.0000***
Portland, OR--WA Urbanized Area	0.25	0.1510
Providence, RI--MA Urbanized Area	3.44	0.0000***
Riverside--San Bernardino, CA Urbanized Area	8.15	0.0000***
Sacramento, CA Urbanized Area	2.76	0.0000***

St. Louis, MO--IL Urbanized Area	0.67	0.0000***
San Antonio, TX Urbanized Area	0.33	0.0537*
San Diego, CA Urbanized Area	3.40	0.0000***
San Francisco--Oakland, CA Urbanized Area	4.87	0.0000***
San Jose, CA Urbanized Area	6.78	0.0000***
Seattle, WA Urbanized Area	1.20	0.0000***
Tampa--St. Petersburg, FL Urbanized Area	-0.46	0.0072***
Virginia Beach, VA Urbanized Area	0.17	0.3362
Model adjusted R^2 = 0.96		
Model *p*-value = 0.0000***		
n = 528		

[a]Income is the mid-point of the Census household income category, transformed by subtracting the mean household income.
[b]The reference UA (for which the UA specific dummy variable = 0) is Atlanta, GA.

Statistical significance: *p<0.1; **p<0.05; ***p<0.01

Table S11. Linear regression model results for population-weighted concentrations for White householders in small Urban Areas

Variable	Coefficient	*p*-value
(Intercept)	9.81	0.0000***
Income[a]	-6.59E-06	0.0000***
Income[a]-squared	3.75E-11	0.0000***
Urban Area-specific dummy variables[b]		
Abilene, TX Urbanized Area	0.49	0.0000***
Akron, OH Urbanized Area	3.22	0.0000***
Albany, GA Urbanized Area	-2.86	0.0000***
Albany, NY Urbanized Area	0.77	0.0000***
Albuquerque, NM Urbanized Area	4.67	0.0000***
Alexandria, LA Urbanized Area	-1.59	0.0000***
Allentown--Bethlehem, PA--NJ Urbanized Area	3.98	0.0000***
Alton, IL Urbanized Area	-0.51	0.0000***
Altoona, PA Urbanized Area	2.78	0.0000***
Amarillo, TX Urbanized Area	1.88	0.0000***
Ames, IA Urbanized Area	-2.95	0.0000***
Anderson, IN Urbanized Area	0.05	0.5850
Anderson, SC Urbanized Area	-1.73	0.0000***
Ann Arbor, MI Urbanized Area	1.17	0.0000***
Anniston, AL Urbanized Area	-3.28	0.0000***
Antioch, CA Urbanized Area	2.71	0.0000***
Appleton, WI Urbanized Area	-0.19	0.0456
[*Continued; 407 total small Urban Areas*]		
Model adjusted $R^2 = 0.98$		
Model *p*-value = 0.0000***		
n = 6512		

[a] Income is the mid-point of the Census household income category, transformed by subtracting the mean household income.
[b]The reference UA (for which the UA specific dummy variable = 0) is Aberdeen, MD.

Statistical significance: *$p<0.1$; **$p<0.05$; ***$p<0.01$

Table S12. Linear regression model results for population-weighted concentrations for Black householders in small Urban Areas

Variable	Coefficient	p-value
(Intercept)	10.15	0.0000***
Income[a]	-6.56E-06	0.0000***
Income[a]-squared	5.13E-11	0.0000***
Urban Area-specific dummy variables[b]		
Abilene, TX Urbanized Area	0.38	0.0611*
Akron, OH Urbanized Area	4.65	0.0000***
Albany, GA Urbanized Area	-2.29	0.0000***
Albany, NY Urbanized Area	2.38	0.0000***
Albuquerque, NM Urbanized Area	4.17	0.0000***
Alexandria, LA Urbanized Area	-0.93	0.0000***
Allentown--Bethlehem, PA--NJ Urbanized Area	5.01	0.0000***
Alton, IL Urbanized Area	-1.04	0.0000***
Altoona, PA Urbanized Area	3.14	0.0000***
Amarillo, TX Urbanized Area	1.90	0.0000***
Ames, IA Urbanized Area	-3.51	0.0000***
Anderson, IN Urbanized Area	0.14	0.4763
Anderson, SC Urbanized Area	-1.52	0.0000***
Ann Arbor, MI Urbanized Area	1.01	0.0000***
Anniston, AL Urbanized Area	-2.26	0.0000***
Antioch, CA Urbanized Area	2.47	0.0000***
Appleton, WI Urbanized Area	-0.63	0.0030**
[*Continued; 407 total small Urban Areas*]		
Model adjusted R^2 = 0.93		
Model p-value = 0.0000***		
n = 5776		

[a]Income is the mid-point of the Census household income category, transformed by subtracting the mean household income.
[b]The reference UA (for which the UA specific dummy variable = 0) is Aberdeen, MD.

Statistical significance: *p<0.1; **p<0.05; ***p<0.01

Table S13. Linear regression model results for population-weighted concentrations for Hispanic householders in small Urban Areas

Variable	Coefficient	*p*-value
(Intercept)	10.07	0.0000***
Income[a]	-6.37E-06	0.0000***
Income[a]-squared	3.90E-11	0.0000***
Urban Area-specific dummy variables[b]		
Abilene, TX Urbanized Area	0.76	0.0029*
Akron, OH Urbanized Area	4.01	0.0000***
Albany, GA Urbanized Area	-2.32	0.0000***
Albany, NY Urbanized Area	1.34	0.0000***
Albuquerque, NM Urbanized Area	3.98	0.0000***
Alexandria, LA Urbanized Area	-1.31	0.0000***
Allentown--Bethlehem, PA--NJ Urbanized Area	5.31	0.0000***
Alton, IL Urbanized Area	-0.53	0.0589*
Altoona, PA Urbanized Area	3.26	0.0000***
Amarillo, TX Urbanized Area	1.78	0.0000***
Ames, IA Urbanized Area	-3.34	0.0000***
Anderson, IN Urbanized Area	-0.15	0.5795
Anderson, SC Urbanized Area	-1.93	0.0000***
Ann Arbor, MI Urbanized Area	1.11	0.0000***
Anniston, AL Urbanized Area	-2.85	0.0000***
Antioch, CA Urbanized Area	2.33	0.0000***
Appleton, WI Urbanized Area	-0.50	0.0585*
[*Continued; 407 total small Urban Areas*]		

Model adjusted $R^2 = 0.90$

Model *p*-value = 0.0000***

n = 5769

[a]Income is the mid-point of the Census household income category, transformed by subtracting the mean household income.
[b]The reference UA (for which the UA specific dummy variable = 0) is Aberdeen, MD.

Statistical significance: *p<0.1; **p<0.05; ***p<0.01

Table S14. Linear regression model results for population-weighted concentrations for Asian householders in small Urban Areas

Variable	Coefficient	*p*-value
(Intercept)	9.80	0.0000***
Income[a]	-7.55E-06	0.0000***
Income[a]-squared	3.22E-11	0.0000***
Urban Area-specific dummy variables[b]		
Abilene, TX Urbanized Area	0.21	0.4886
Akron, OH Urbanized Area	3.99	0.0000***
Albany, GA Urbanized Area	-2.61	0.0000***
Albany, NY Urbanized Area	1.19	0.0000***
Albuquerque, NM Urbanized Area	4.95	0.0000***
Alexandria, LA Urbanized Area	-0.97	0.0019**
Allentown--Bethlehem, PA--NJ Urbanized Area	4.40	0.0000***
Alton, IL Urbanized Area	-0.08	0.8418
Altoona, PA Urbanized Area	2.67	0.0000***
Amarillo, TX Urbanized Area	0.98	0.0000***
Ames, IA Urbanized Area	-3.34	0.0000***
Anderson, IN Urbanized Area	0.19	0.6219
Anderson, SC Urbanized Area	-1.48	0.0000***
Ann Arbor, MI Urbanized Area	1.34	0.0000***
Anniston, AL Urbanized Area	-3.52	0.0000***
Antioch, CA Urbanized Area	2.65	0.0000***
Appleton, WI Urbanized Area	0.20	0.4798
[*Continued; 407 total small Urban Areas*]		

Model adjusted $R^2 = 0.86$

Model p-value = 0.0000***

$n = 5192$

[a]Income is the mid-point of the Census household income category, transformed by subtracting the mean household income.
[b]The reference UA (for which the UA specific dummy variable = 0) is Aberdeen, MD.

Statistical significance: *$p<0.1$; **$p<0.05$; ***$p<0.01$

Table S15. Linear regression model results for population-weighted concentrations for White householders in rural areas

Variable	Coefficient	*p*-value
(Intercept)	4.54	0.0000***
Income[a]	3.74E-06	0.0000***
Income[a]-squared	-2.35E-11	0.0000***
Model adjusted R^2 = 0.98		
Model *p*-value = 0.0000***		
n = 16		

[a]Income is the mid-point of the Census household income category, transformed by subtracting the mean household income.

Statistical significance: **p*<0.1; ***p*<0.05; ****p*<0.01

Table S16. Linear regression model results for population-weighted concentrations for Black householders in rural areas

Variable	Coefficient	*p*-value
(Intercept)	3.76	0.0000***
Income[a]	2.47E-06	0.0000***
Income[a]-squared	-1.92E-11	0.0017**
Model adjusted R^2 = 0.73		
Model *p*-value = 0.0000***		
n = 16		

[a]Income is the mid-point of the Census household income category, transformed by subtracting the mean household income.

Statistical significance: *p<0.1; **p<0.05; ***p<0.01

Table S17. Linear regression model results for population-weighted concentrations for Hispanic householders in rural areas

Variable	Coefficient	*p*-value
(Intercept)	5.79	0.0000***
Income[a]	-3.06E-06	0.0000***
Income[a]-squared	1.02E-11	0.1280
Model adjusted R^2 = 0.79		
Model *p*-value = 0.0000***		
n = 16		

[a]Income is the mid-point of the Census household income category, transformed by subtracting the mean household income.

Statistical significance: *$p<0.1$; **$p<0.05$; ***$p<0.01$

Table S18. Linear regression model results for population-weighted concentrations for Asian householders in rural areas

Variable	Coefficient	*p*-value
(Intercept)	4.865	0.0000***
Income[a]	3.77E-06	0.0029**
Income[a]-squared	-2.62E-11	0.0638*
Model adjusted R^2 = 0.46		
Model *p*-value = 0.0072***		
n = 16		

[a]Income is the mid-point of the Census household income category, transformed by subtracting the mean household income.

Statistical significance: *p<0.1; **p<0.05; ***p<0.01

Table S19. Linear regression model results for mean Block group concentrations for the high percent White tertile in large Urban Areas

Variable	Coefficient	*p*-value
(Intercept)	31.80	0.0000***
Income[a]	1.56E-06	0.0000***
Income[a]-squared	2.33E-10	0.0000***
Percent White	-0.17	0.0000***
Model adjusted R^2 = 0.03		
Model *p*-value = 0.0000***		
n = 13,632		

[a]Income is the mean Block Group income, transformed by subtracting the mean household income.

Statistical significance: *p<0.1; **p<0.05; ***p<0.01

Table S20. Linear regression model results for mean Block Group concentrations for the medium percent White tertile in large Urban Areas

Variable	Coefficient	*p*-value
(Intercept)	23.69	0.0000***
Income[a]	2.89E-05	0.0000***
Income[a]-squared	-1.03E-11	0.0000***
Percent White	-0.07	0.0000***
Model adjusted R^2 = 0.03		
Model *p*-value = 0.0000***		
n = 13,633		

[a]Income is the mean Block Group income, transformed by subtracting the mean household income.

Statistical significance: *p<0.1; **p<0.05; ***p<0.01

Table S21. Linear regression model results for mean Block Group concentrations for the low percent White tertile in large Urban Areas

Variable	Coefficient	*p*-value
(Intercept)	23.22	0.0000***
Income[a]	-9.61E-05	0.0000***
Income[a]-squared	4.28E-10	0.0000***
Percent White	-0.07955	0.0000***
Model adjusted R^2 = 0.02		
Model *p*-value = 0.0000***		
n = 13,632		

[a]Income is the mean Block Group income, transformed by subtracting the mean household income.

Statistical significance: *p<0.1; **p<0.05; ***p<0.01

Table S22. Linear regression model results for Block Group concentrations for the high percent White tertile in medium Urban Areas

Variable	Coefficient	*p*-value
(Intercept)	19.53	0.0000***
Income[a]	-3.70E-05	0.0000***
Income[a]-squared	8.01E-10	0.0000***
Percent White	-0.08	0.0000***

Model adjusted R^2 = 0.02

Model *p*-value = 0.0000***

n = 12,787

[a]Income is the mean Block Group income, transformed by subtracting the mean household income.

Statistical significance: *p<0.1; **p<0.05; ***p<0.01

Table S23. Linear regression model results for Block Group concentrations for the medium percent White tertile in medium Urban Areas

Variable	Coefficient	*p*-value
(Intercept)	18.99	0.0000***
Income[a]	3.45E-05	0.0000***
Income[a]-squared	1.90E-11	0.7670
Percent White	-0.07	0.0000***
Model adjusted R^2 = 0.04		
Model *p*-value = 0.0000***		
n = 12,787		

[a]Income is the mean Block Group income, transformed by subtracting the mean household income.

Statistical significance: *p<0.1; **p<0.05; ***p<0.01

Table S24. **Linear regression model results for Block Group concentrations for the low percent White tertile in medium Urban Areas**

Variable	Coefficient	*p*-value
(Intercept)	15.60	0.0000***
Income[a]	-5.16E-05	0.0000***
Income[a]-squared	3.61E-09	0.0000***
Percent White	-0.01	0.0000***

Model adjusted R^2 = 0.01

Model *p*-value = 0.0000***

n = 12,787

[a]Income is the mean Block Group income, transformed by subtracting the mean household income.

Statistical significance: *p<0.1; **p<0.05; ***p<0.01

Table S25. Linear regression model results for Block Group concentrations for the high percent White tertile in small Urban Areas

Variable	Coefficient	*p*-value
(Intercept)	15.51	0.0000***
Income[a]	-8.02E-06	0.0002***
Income[a]-squared	4.82E-11	0.0019***
Percent White	-0.06	0.0000***
Model adjusted R^2 = 0.005		
Model *p*-value = 0.0000***		
n = 13,372		

[a]Income is the mean Block Group income, transformed by subtracting the mean household income.

Statistical significance: *p<0.1; **p<0.05; ***p<0.01

Table S26. Linear regression model results for Block Group concentrations for the medium percent White tertile in small Urban Areas

Variable	Coefficient	*p*-value
(Intercept)	15.40	0.0000***
Income[a]	1.68E-05	0.0000***
Income[a]-squared	3.58E-10	0.0039***
Percent White	-0.06	0.0000***

Model adjusted R^2 = 0.02

Model *p*-value = 0.0000***

n = 13,371

[a]Income is the mean Block Group income, transformed by subtracting the mean household income.

Statistical significance: *p<0.1; **p<0.05; ***p<0.01

Table S27. Linear regression model results for Block Group concentrations for the low percent White tertile in small Urban Areas

Variable	Coefficient	*p*-value
(Intercept)	12.20	0.0000***
Income[a]	-4.99E-06	0.3671
Income[a]-squared	4.00E-10	0.0522*
Percent White	-0.01	0.0000***
Model adjusted R^2 = 0.02		
Model *p*-value = 0.0000***		
n = 13,372		

[a]Income is the mean Block Group income, transformed by subtracting the mean household income.

Statistical significance: **p*<0.1; ***p*<0.05; ****p*<0.01

Table S28. **Linear regression model results for Block Group concentrations for the high percent White tertile in rural areas**

Variable	Coefficient	*p*-value
(Intercept)	1.68	0.3600
Income[a]	9.74E-05	0.0000***
Income[a]-squared	-8.08E-10	0.0000***
Percent White	0.04	0.0475**
Model adjusted R^2 = 0.005		
Model *p*-value = 0.0000***		
n = 24,588		

[a]Income is the mean Block Group income, transformed by subtracting the mean household income.

Statistical significance: *p<0.1; **p<0.05; ***p<0.01

Table S29. Linear regression model results for Block Group concentrations for the medium percent White tertile in rural areas

Variable	Coefficient	*p*-value
(Intercept)	15.07	0.0000***
Income[a]	1.02E-04	0.0000***
Income[a]-squared	-5.66E-10	0.0000***
Percent White	-0.09	0.0000***
Model adjusted R^2 = 0.11		
Model *p*-value = 0.0000***		
n = 24,588		

[a]Income is the mean Block Group income, transformed by subtracting the mean household income.

Statistical significance: *p<0.1; **p<0.05; ***p<0.01

Table S30. Linear regression model results for Block Group concentrations for the low percent White tertile in rural areas

Variable	Coefficient	*p*-value
(Intercept)	9.44	0.0000***
Income[a]	1.45E-04	0.0000***
Income[a]-squared	-5.30E-10	0.0000***
Percent White	-0.04	0.0000***
Model adjusted R^2 = 0.08		
Model *p*-value = 0.0000***		
n = 24,588		

[a]Income is the mean Block Group income, transformed by subtracting the mean household income.

Statistical significance: *p<0.1; **p<0.05; ***p<0.01
